# Viable intrauterine pregnancy after spontaneous bilateral tubal ectopics in a multiparous woman: a case report

**DOI:** 10.1186/1752-1947-7-159

**Published:** 2013-06-20

**Authors:** Sreeja Rani V R, Geetha Puliyath

**Affiliations:** 1 Department of Obstetrics and Gynaecology, SUT Academy of Medical Sciences, Trivandrum, Kerala, India

**Keywords:** Bilateral, Ectopic, Pregnancy, Salpingostomy, Tubal

## Abstract

**Introduction:**

Bilateral ectopic pregnancies are increasing due to an increase in the incidence of pelvic inflammatory disease and increase in assisted reproductive techniques. Spontaneous conception after bilateral ectopic pregnancies is extremely rare.

**Case presentation:**

A 33-year-old Indian woman presented with ruptured ectopic pregnancy associated with hemoperitoneum for which she underwent a laparotomy. The diagnosis of bilateral ectopic pregnancies was made during surgery. Salpingectomy was done on one side and the other tube was conserved with salpingostomy. Six months after surgery, she conceived spontaneously and a transvaginal ultrasound examination revealed a 5-weeks live intrauterine pregnancy.

**Conclusion:**

A meticulous surgical technique conserving one fallopian tube resulted in subsequent viable intrauterine pregnancy. The approach to such a case along with steps to minimize tubal damage is presented.

## Introduction

In the past few decades the incidence of unusual presentations of ectopic pregnancies has risen along with an increase in assisted reproductive techniques [1]. Spontaneous conception after bilateral tubal ectopic pregnancy is associated with an increased risk of recurrent ectopic pregnancy. It calls for maximum care to prevent tubal damage when conservative surgery is done on the fallopian tube.

## Case presentation

A 33-year-old Indian woman presented with irregular and scanty bleeding with occasional abdominal pain for 17 days. There was no history of missed periods and all her previous cycles were regular with normal flow. She has had two full-term vaginal deliveries. The first baby died 52 days after delivery due to pneumonia and the second child is healthy and 7-years old. She has a history of a intrauterine copper contraceptive device insertion 10 months after her second delivery, which was removed 8 months before her presenting symptoms. She was sexually active and not using any contraception for the past 8 months. There was no history suggestive of pelvic inflammatory disease, previous laparotomy or intake of fertility-enhancing drugs.

On examination, the patient was pale, although her vitals were stable. Her abdomen was soft; there was no tenderness or guarding. A pelvic examination revealed mild bleeding per vagina, a normal-sized uterus and left forniceal tenderness. Her urine pregnancy test was positive. With the clinical suspicion of ectopic pregnancy, an emergency ultrasound examination was performed which showed an empty normal-sized uterus and a gestational sac of 5×4cm with a fetal pole having cardiac activity in the left fallopian tube. There was also a moderate amount of fluid in the pouch of Douglas.

**Figure 1:**
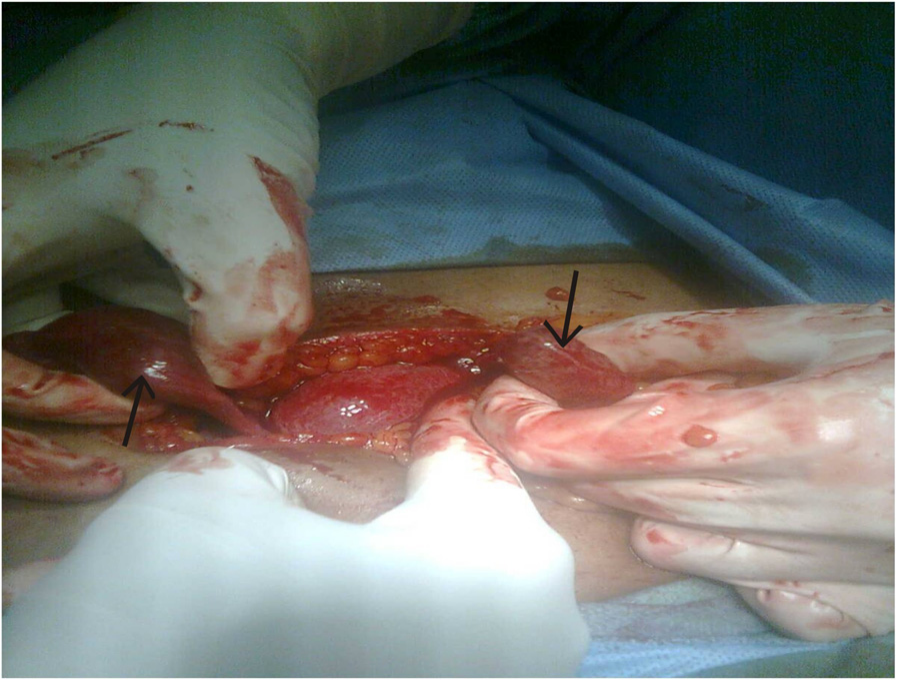
Picture showing uterus in the middle and two arrows indicating the ectopic pregnancies on both the fallopian tubes.

In view of hemoperitoneum, an emergency laparotomy was done with informed consent. On opening, there was about 300mL of blood in the peritoneal cavity and the ampullary region of the left tube was distended with a 5×4cm mass. Fresh blood was dripping from the fimbrial end of the left fallopian tube. The right fallopian tube was also distended at the ampullary region with a 3×3cm mass. With these findings, a bilateral ampullary pregnancy with features of tubal abortion on the left fallopian tube was diagnosed (Figure 1). The intraoperative findings were explained to the woman’s husband and the possibilities of salpingectomy or salpingostomy were discussed. The risk of recurrent ectopic pregnancy and effects on future fertility were explained. Because the left tube was extensively damaged by the ectopic gestation and hematoma, salpingectomy was done. A linear salpingostomy was performed on her right fallopian tube after ligating the feeding vessel on the mesosalpinx 1cm below the ampullary ectopic pregnancy to control bleeding. The products of conception were removed. The use of cautery was minimal.

**Figure 2:**
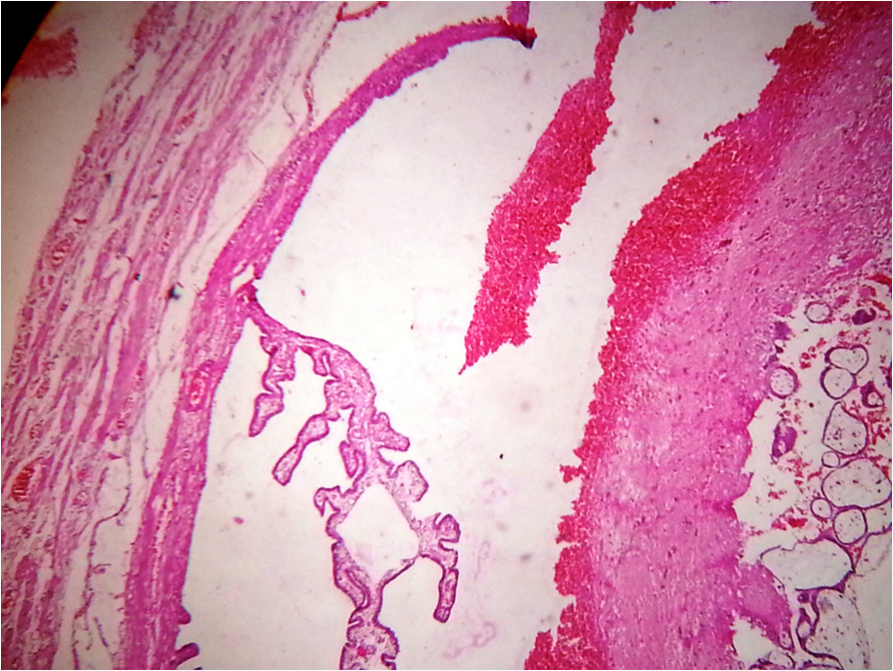
Section of the left fallopian tube with chorionic villi inside and fibrin deposition around the villi.

**Figure 3:**
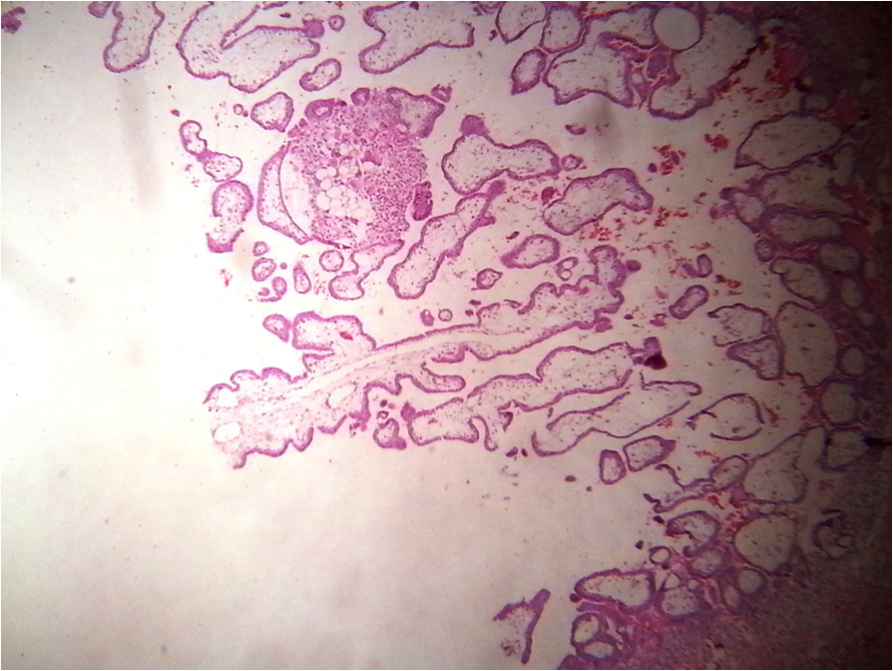
Chorionic villi from the right tube.

The products from the right tube and cut portion of the left tube with ectopic pregnancy were sent for histopathological examination which showed presence of chorionic villi on both sides and the diagnosis of bilateral ampullary ectopic pregnancy was confirmed (Figures 2 and 3). The postoperative period was uneventful and the patient was discharged on the fifth postoperative day.

Serum beta human chorionic gonadotropin (HCG) became undetectable 3 weeks after surgery and the woman was advised to report missed periods or irregular bleeding immediately. Seven months later, she came with missed periods and a urine pregnancy test was positive. A serum beta HCG estimation and a transvaginal ultrasound examination at 5 weeks revealed a single intrauterine pregnancy with good fetal cardiac activity. The right tube and both ovaries were normal. The patient continued her pregnancy and eventually delivered a healthy baby at term.

## Discussion

Bilateral tubal ectopic pregnancy is the rarest form of ectopic pregnancy [2]. The incidence is reported to be five in one million deliveries [3]. Many cases are as a result of assisted reproductive techniques.

The postulated mechanisms of bilateral ectopic pregnancy include multiple ovulation, transperitoneal migration of trophoblastic tissue from one tube to another or superfetation [4]. There are case reports on bilateral tubal pregnancy diagnosed with a ruptured ectopic on one tube and chronic ectopic on the other [5]. The criteria for diagnosis of bilateral ectopic pregnancy were first suggested by Fishback and later revised by Norris who stated that microscopic demonstration of chorionic villi in each tube was sufficient for the diagnosis [6].

The preoperative diagnosis of bilateral ectopic pregnancy remains a challenge. Serum beta HCG estimation is not reliable as the values will be elevated more than that of a single ectopic. Detection with ultrasound scan is difficult and only very few cases have been diagnosed preoperatively by ultrasound [7].

There are very few case reports in the literature on medical management of bilateral ectopic pregnancies. However, one report showed successful treatment of bilateral ectopics by two consecutive methotrexate injections into the gestational sac under vaginal ultrasonographic guidance [8]. Another report shows failure of single dose methotrexate given for a single ectopic pregnancy which was later diagnosed to be a bilateral ectopic pregnancy [9]. There is also a report on failed conservative management for suspected single ectopic pregnancy which on laparotomy was found to be a bilateral tubal ectopic pregnancy [10].

During surgery for ectopic pregnancy, inspection of both tubes, ovaries and peritoneal cavity should be done. There are reports of failure to identify one of the bilateral ectopic pregnancies and the patient presenting a few days after surgery with acute abdominal pain [11]. Management options should be discussed with the patient and relatives because of increased risk of recurrent ectopic pregnancy and future infertility.

The fallopian tube derives its blood supply from branches of uterine and ovarian arteries. With an ectopic gestation in the tube, prominent blood vessels are seen in the mesosalpinx supplying the site of the ectopic pregnancy. Ligation of the main vessel of supply before removal of the products of conception by salpingostomy helps in decreasing bleeding from the site of the ectopic pregnancy. It reduces the use of cautery with less damage to the fallopian tubes.

Only two previous reported cases of intrauterine conception following surgery done for bilateral ectopic pregnancy were retrieved after an extensive database search [12,13]. They were similar to the present case discussed, in that only one tube was conserved during surgery. After bilateral spontaneous ectopics, there is a higher risk for heterotopic pregnancies as the patient has twin proneness [14].

Bilateral ectopic pregnancies are being managed in different ways. There are case reports where both tubes have been conserved [15]. No guidelines are presently available on this topic.

## Conclusions

The present case and two other cases of intrauterine conception after bilateral ectopic pregnancies which have been studied show conservation of only a single fallopian tube [12,13]. Although the surgical approach was laparotomy, studies have shown that conservation of future fertility and risk of recurrent ectopic pregnancy are the same in laparoscopy and laparotomy. The tubal damage may significantly be reduced by ligating the main feeding vessel in the mesosalpinx prior to salpingostomy.

## Consent

Written informed consent was obtained from the patient for publication of this case report and accompanying images. A copy of the written consent is available for review by the Editor-in-Chief of this journal.

## Competing interests

The authors declare that they have no competing interests.

## Authors’ contributions

GP analyzed and interpreted the patient data regarding diagnosis and contributed in writing the manuscript. SR performed the surgery and was a major contributor in writing the manuscript. Both authors read and approved the final manuscript.
